# Supplementary material for: Identification of photoperiod-induced specific miRNAs in the adrenal glands of Sunite sheep (Ovis aries)
Source: Front Vet Sci. 2022 Jul 22;9:888207. doi: 10.3389/fvets.2022.888207 (PMC9354845; doi:10.3389/fvets.2022.888207)
Supplement: Supplementary file 1 [file Data_Sheet_1.ZIP › Supplementary file/miRDeep2 file.zip!/miRDeep2 file/Know_Structure/SPLP42A3/oar-mir-136.pdf]

miRBase precursor : oar-mir-136
Total read count : 5546
oar-miR-136 read count : 1973
remaining reads : 3573

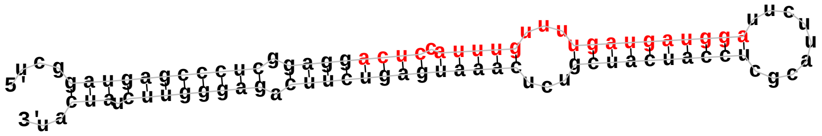

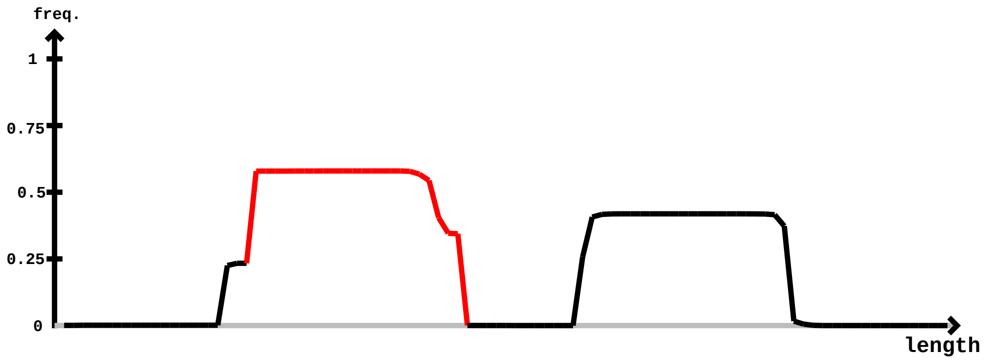

oar-miR-136

5'- ucggaugagcccucggaggacuccauuuguuuugaugauggauucuuacgcuccaucaucgucucaaaugagucuucagaggguucuaucau -3'	exp		
...(((((((((((.((((((((.(((((...((((((((((.........))))))))))...))))))))))))).)))))))).)))..	reads	mm	sample
ucggaugagcccucggag..	1	0	seq
ucggaugagcccucggagg...	3	0	seq
.cggaugagcccucggagg...	3	0	seq
................aggacuccauuuguuuuga...	7	0	seq
................aggacuccauuuguuuugau..	58	0	seq
................aggacuccauuuguuuugaug...	132	0	seq
................aggacuccauuuguuuugauga..	760	0	seq
................aggacuccauuuguuuugaugau...	284	0	seq
................aggacuccauuuguuuugaugaug..	1	0	seq
.................ggacuccauuuguuuugauga..	10	0	seq
.................ggacuccauuuguuuugaugau...	36	0	seq
..................gacuccauuuguuuugaugaugg...	1	0	seq
...................Ccuccauuuguuuugau..	1	1	seq
...................Ccuccauuuguuuugauga..	1	1	seq
...................acuccauuuguuuugauga..	1	0	seq
...................acuccauuuguuuugaugau...	6	0	seq
...................acuccauuuguuuugaugaug..	9	0	seq
...................acuccauuuguuuugaugaugg...	1904	0	seq
....................cuccauuuguuuugaugaugg...	1	0	seq
.......................cauuuguuuugaugaugg...	1	0	seq
.......................cauuuguuuugaugaugga..	1	0	seq
..........................uuguuuugaugauggauucu..	1	0	seq
....caucaucgucucaaaug......................	1	0	seq
....caucaucgucucaaauga.....................	1	0	seq
....caucaucgucucaaaugag....................	2	0	seq
....caucaucgucucaaaugagu...................	10	0	seq
....caucaucgucucaaaugaguc..................	178	0	seq
....caucaucgucucaaaugagucu.................	1227	0	seq
....caucaucgucucaaaugagucuu................	8	0	seq
....caucaucgucucaaaugagucuuc...............	1	0	seq
....aucaucgucucaaaugagu...................	5	0	seq
....aucaucgucucaaaugaguc..................	58	0	seq
....aucaucgucucaaaugagucu.................	738	0	seq
....aucaucgucucaaaugagucuu................	27	0	seq
....aucaucgucucaaaugagucuuc...............	1	0	seq

oar-miR-136

```
ucggaugagcccucggaggacuccauuuguuuugaugauggauucuuacgcuccaucaucgucucaaaugagucuucagaggguucuaucau
.......................................................ucaucgucucaaaugagucu.................    11    0    seq
.......................................................ucaucgucucaaaugagucuu................    23    0    seq
.......................................................ucaucgucucaaaugagucuuc...............    17    0    seq
.......................................................ucaucgucucaaaugagucuuca..............    4     0    seq
........................................................caucgucucaaaugagucu.................    1     0    seq
........................................................caucgucucaaaugagucuu................    1     0    seq
........................................................caucgucucaaaugagucuuc...............    7     0    seq
........................................................caucgucucaaaugagucuuca..............    1     0    seq
.........................................................aucgucucaaaugagucuu................    2     0    seq
```
